# Effect of pharmacist-led medication reviews on appropriateness of prescribing in patients with dementia – results from the cluster randomized controlled “DemStepCare” study

**DOI:** 10.1186/s12877-025-06565-6

**Published:** 2025-11-04

**Authors:** Marie Kocklaeuner, Katharina Geschke, Andreas Fellgiebel, Irene Kraemer

**Affiliations:** 1 Department of Pharmacy, University Medical Center, Mainz, Germany; 2 Department of Psychiatry and Psychotherapy, University Medical Center, Mainz, Germany; 3 Hospital for Psychiatry, Psychosomatic and Psychotherapy, Agaplesion Elisabethenstift, Darmstadt, Germany

**Keywords:** Dementia patient, Pharmacist-led medication review, Drug-related problem, Medication appropriate index, Controlled study

## Abstract

**Background:**

Patients with dementia (PwD) are often geriatric patients treated with multimedication increasing the risk of drug-related problems (DRP) and inappropriate prescriptions. Pharmacist-led medication reviews are effective to solve DRPs and to improve medication appropriateness. The Medication Appropriateness Index (MAI) is a reliable, valid and standardised tool for detecting multiple dimensions of inappropriate prescribing. The research aimed to evaluate the impact of pharmacist-led medication reviews on medication appropriateness of PwDs as part of the DemStepCare study, using the MAI to categorise potentially inappropriate prescriptions.

**Methods:**

Comprehensive medication reviews were performed for PwD treated as outpatients over 39 months in the longitudinal, cluster-randomized controlled DemStepCare study by a clinical pharmacist utilizing the project-specific, electronic patient record. Modified MAI scores were calculated at baseline and over the course of the study in the intervention group (IG) and control group (CG) at predefined time points. Only in the interventional arm, responsible GPs were informed about the result of the medication review and the pharmacist’s recommendation to improve medication appropriateness.

**Results:**

198 PwDs were enrolled in the IG and 47 PwDs in the CG (intention-to-treat collective). For IG patients, the modified MAI sum (SS) and patient score (PS) decreased significantly (*p* < 0.001) until t_1_, i.e. over the first 9 or 11 months of the study (baseline: SS = 15.24 ± 12.78; PS = 2.10 ± 1.53 / t_1_: SS = 7.50 ± 9.54; PS = 0.97 ± 1.13). The number of DRPs in this period was significantly reduced as well.

**Conclusion:**

Even a single comprehensive medication review significantly improved medication appropriateness in PwD. Based on the results, pharmacist-led medication reviews are strongly recommended for PwDs.

**Supplementary Information:**

The online version contains supplementary material available at 10.1186/s12877-025-06565-6.

## Background

 Multimorbidity is generally defined as the presence of more than two chronic diseases [1] and is often combined with polypharmacy. In Germany, the resulting polypharmacy is determined as simultaneous and long-term use of five or more medications [2]. The complexity of polypharmacy in multimorbid geriatric patients and the approach of deprescribing specified medications is already addressed in several guidelines [3–5]. The diagnosis of dementia is often associated with increased age, co-morbidities and polypharmacy [6, 7]. On average, each patient with dementia takes 5–10 medications per day [8–10]. As the number of medication errors is positively related to the number of medications used [11] and due to the frequent use of centrally acting drugs (e.g., psychotropics, opioids) [12], patients with dementia (PwD) are particularly at risk for drug-related problems (DRP) [13, 14] and reduced medication safety [15]; this can lead to complex interventions and increasing healthcare costs [16]. Progress of dementia induces the prescription of additional medication with often questionable benefit [17] and increased frequency of medication switches, especially when behavioural and psychological symptoms are present [18].

Pharmaceutical interventions are intended to solve the identified DRPs and to improve medication appropriateness and safety [19, 20]. Clinical pharmacists are predestined to reduce frequency and duration of DRPs by pharmaceutical interventions, since they know the patient’s medical record and clinical condition [21]. Numerous studies show that pharmacist-led medication reviews can improve quality and safety of medication therapy [22–24]. To measure the impact of pharmaceutical interventions, the Medication Appropriateness Index (MAI) [25, 26] was established and scientifically approved [27]. Other acknowledged explicit tools comprise specific issues, like potentially inappropriate medication in old patients (PIM), deprescribing and underuse [28].

To our best knowledge, the impact of pharmacist-led medication reviews on medication safety in PwD living at home has not been evaluated in clinical studies yet. The aim of our study was to investigate the effectiveness of pharmacist-led medication reviews on the appropriateness and safety of pharmacotherapy in PwD.

## Methods

### Design

The study was an integrated part of DemStepCare, a longitudinal, cluster-randomised controlled study, lasting from April 2019 to March 2023 [29]. Participating general practitioners (GP) were randomised by an independent trust centre into the intervention group (IG) and the control group (CG). PwD in the CG received outpatient treatment according to standard care [29], whereas IG patients were taken care by a case manager, had access to a crisis outpatient clinic when needed, and received pharmacy-led medication reviews communicated to the GP via electronic patient record [29].

The study protocol was approved by the ethics committee of the Medical Chamber of Rhineland-Palatinate (reference no. 2019–14427). Investigations were conducted in line with the Declaration of Helsinki and principles outlined in recommendations for Good Clinical Practice and Good Epidemiological Practice. Written informed consent was obtained from each participant before inclusion in the study. The trial was registered in the German Clinical Trials Register (DRKS00023560) on 2020.11.13.

### Setting and study population

The GPs participating were located in Rhineland-Palatinate. They recruited PwD from October 2019 to March 2022. Inclusion criteria were: (i) confirmed or suspected dementia (F00-F03), (ii) treatment by a GP included in the study, (iii) member of statutory health insurance, and (iv) written informed consent to participate in the study. Exclusion criteria were: (i) dementia not confirmed, (ii) patient residence ≥ 10 km outside the study area, (iii) living in a residential care facility, (iv) member of private health insurance, (v) patients insured according to § 264 SGB V. Age was irrelevant for inclusion or exclusion.

#### Medication reviews

Medication reviews were performed by a clinical pharmacist based on the electronic patient record ISPC (Smart-Q Softwaresysteme GmbH Bochum, Germany) provided by the GP and the case manager. ISPC was especially implemented for the DemStepCare project [29] and comprises data as age, sex, diagnoses, laboratory reports, vital signs, medication, allergies, and treatment adherence.

Table 1 shows the time points at which medications reviews were performed. Due to the COVID-19 pandemic, the study was suspended for two months. Hence, medication reviews for patients enrolled before the interruption, had to be postponed from month 9 to month 11 (see t_1_ in Table 1). Consequently, for patients in the IG the Q4 medication review was omitted.

**Table 1:** Time points of medication reviews

Time point of medication review		
Abbreviation	Intervention Group	Control Group
t_0_	Time point of enrolment, baseline	Time point of enrolment, baseline
t_1_	Month 9 or 11 after enrolment (delay related to COVID-19 pandemic)	Month 9 or 11 after enrolment (delay related to COVID-19 pandemic)
t_2_	Patient individual end of study, max. 39 months after enrolment	Patient individual end of study, max. 39 months after enrolment
Q1 to Qx	Quarterly during the study period	No additional time points

Medication appropriateness was assessed via the Medication Appropriateness Index (MAI), a reliable, valid and standardised tool for measuring potentially inappropriate prescribing in patients with polypharmacy [25, 26]. The score consists of ten criteria, i.e. questions relevant for appropriate prescribing (see Supplement 1). The criterion ‘cost-effectiveness of prescribing’ was omitted, resulting in a modified MAI (hereinafter referred to as MAI). Each criterion was scored on a 3-point scale (1 = appropriate, 2 = marginally appropriate, 3 = inappropriate) [25], with the scores dichotomised and weighted according to the publication of Samsa et al. [26]. Thereby, criteria rated as appropriate or marginally appropriate were assigned 0 points. Criteria rated as inappropriate, were assigned the calculated weighted score. The rating was done by a clinical pharmacist. To check for possible subjectivity, control samples were reviewed by a second clinical pharmacist. All ambiguous ratings and any resulting questions were discussed. The MAI score was defined as the sum of points given for each criterion per medication (maximum score 17). Thus, inappropriateness of prescription was positively correlated with the MAI score. The MAI sum score of an individual patient was then calculated by summing up the MAI score of each prescribed medication. The MAI patient score was calculated by dividing the MAI sum score by the number of medications prescribed. Medication appropriateness and safety was further assessed by several explicit tools:

- Prevalence of DRP according to the PCNE [30].
- Prevalence of potentially inadequate medication (PIM) in patients ≥ 65 years according to the German Priscus list [31], the FORTA (Fit for The Aged) list [32] and STOPP (screening tool of older person’s prescription) criteria [33].
- Prevalence of high-risk medication in PwD. Medication with an inappropriate risk-benefit-balance was identified in an upfront literature review and included allopurinol, anticholinergics, antipsychotics for the treatment of behavioural and psychological symptoms in dementia, benzodiazepines, bisphosphonates, calcium, proton pump inhibitors, sulfonylureas, statins, and high-dose vitamin D.
- Prevalence of underused medication according to the START (screening tool to alert to right treatment) criteria [33].

Only for patients in the IG, results of the medication reviews were communicated to the GP via the electronic patient record. These pharmaceutical recommendations served as a ‘to-do’ for the GP and were also documented in the medical history, which is always available for all members of the interdisciplinary caretaking team. Acceptance and application of pharmaceutical recommendations by the GP were tracked according to modified prescriptions. Voluntarily, the GP could document acceptance or non-acceptance of the pharmaceutical recommendations.

##### Study endpoints

The primary objective of the study was to investigate the impact of pharmacist-led medication reviews on medication appropriateness in PwD. Medication appropriateness was determined by the modified MAI sum and patient score of patients enrolled in the IG and CG.

Secondary endpoints were the prevalence of number of DRP according to PCNE [30], the number of prescribed high risk medications, in geriatric patients ≥ 65 years the number of PIM according to the PRISCUS list [31], FORTA list [32], and STOPP criteria [33], missing medication for untreated diagnosis according to the START criteria [33].

###### Analysis

Statistical analyses were performed using SPSS^®^ Version 27 for Windows^®^ (IBM SPSS Statistics 27). Socio-demographic characteristics of the study population were calculated as absolute and relative rates for categorical and as mean ± standard deviation for continuous characteristics. Data were analysed for the intention-to-treat collective (ITT).

The effect of pharmacist led medication reviews on inappropriate prescribing was determined by comparing the mean modified MAI sum and patient score of patients in the IG and CG at t_0_, t_1_, t_2_. Additionally, the effect of quarterly medication reviews in the IG was analysed over the whole study period. The significance level was set at 5%. Bonferroni adjustment was used to adjust significance levels for multiple testing in the IG over the course of treatment. Results are presented with 95% confidence intervals (95% CI) and effect size Cohen’s d. Since sample size in DemStepCare was not calculated related to inappropriate prescribing, reported p-values are descriptive. The Welch t-test was used for each statistical analysis when the effects of medication reviews were compared between the IG and CG. The Friedman test was used for each statistical analysis of intra group effects in IG patients.

## Results

### Characteristics of the study population

A total of 23 GP offices participated in the IG, recruiting 198 PwD, and 17 GP offices participated in the CG, recruiting 47 PwD.

The socio-demographic and clinical characteristics of the ITT population at t_0_ are given in Table 2. In both groups, PwD suffered primarily from Alzheimer and vascular dementia; most patients had more than one chronic disease diagnosed (types see Table 2) and were taking more than five medications on average.

**Table 2:** Sociodemographic and clinical characteristics of the intention-to-treat population

	Intervention Group (IG) *n* = 198	Control Group (CG) *n* = 47
Gender [% (*n*)]		
Male	38.4 (76)	44.7 (21)
Female	61.6 (122)	55.3 (26)
Age (years)		
M ± SD	81.7 ± 6.4	80.3 ± 7.4
Illness burden (number of documented diseases)		
M ± SD	13.9 ± 7.4	12.0 ± 6.3
Patients with Chronic disease		
Essential hypertension [% (n)]	77.8 (154)	78.7 (37)
Mental and behavioural disorders excl. dementia [% (n)]	52 (103)	44.7 (21)
Diabetes mellitus [% (n)]	37.9 (75)	31.9 (15)
Renal failure [% (n)]	35.4 (70)	14.9 (7)
Risk of falling [% (n)]	32.8 (65)	14.9 (7)
Patients with Diagnosis of dementia		
Dementia due to Alzheimer disease (F00) [% (n)]	42.2 (84)	34 (16)
Vascular dementia (F01) [% (n)]	43.9 (87)	48.9 (23)
Dementia due to other diseases classified elsewhere (F02) [% (n)]	1.5 (3)	12.8 (6)
Unspecified dementia (F03) [% (n)]	12.1 (24)	4.3 (2)
Number of medicines documented by GP		
M ± SD	7.3 ± 3.6	6.9 ± 3.1
Number of antipsychotics prescribed		
≥ 1 antipsychotic [% (n)]	25.3 (50)	25.5 (12)
≥ 2 antipsychotics [% (n)]	6.6 (13)	4.3 (2)
Patients Multimorbidity, Polypharmacy		
Multimorbidity (> 2 chronic diseases) [% (n)]	97.5 (193)	93.6 (44)
Polypharmacy (≥ 5 prescribed medications) [% (n)]	80.3 (159)	78.7 (37)

*Abbreviationsn*–number, *M*–mean value, *SD*–standard deviation

### Prevalence of potentially inappropriate medication according to MAI

Between October 1 st 2019 and December 31 st 2022, a total of 1568 and 119 medication reviews were performed on PwD in the IG and CG, respectively. The prevalence of potentially inappropriate prescriptions related to the nine MAI criteria and assessed on the predefined time points (t_0_, t_1_, t_2_) are given in Table 3. In the IG, potentially inappropriate prescriptions decreased from t_0_ to t_1_ for each MAI criterion, mainly for the criteria “Incorrect intake instructions”, “Inappropriate Indication” and “Lacking Effectiveness”. The average decrease amounted to 5.8%. In the CG, potentially inappropriate prescriptions rates remained mostly unchanged.

**Table 3:** Percentage rates and numbers and of potentially inappropriate prescriptions according to the MAI criteria

MAI criterion	Prevalence of potentially inappropriate prescriptions [% (*n*)]					
	Intervention Group			Control Group		
	t_0_ (*n* = 1435)	t_1_ (*n* = 1135)	t_2_ (*n* = 1084)	t_0_ (*n* = 322)	t_1_ (*n* = 270)	t_2_ (*n* = 264)
Inappropriate Indication	14.1 (202)	5.1 (58)	4.4 (48)	18.9 (61)	18.1 (49)	17.4 (46)
Lacking Effectiveness	14.8 (212)	5.5 (62)	4.9 (53)	20.5 (66)	20.0 (54)	19.3 (51)
Wrong Dosage	10.5 (150)	5.9 (67)	5.4 (58)	9.0 (29)	10.0 (27)	8.7 (23)
Incorrect intake instructions	17 (244)	6.9 (78)	7.0 (76)	15.5 (50)	13.3 (36)	14 (37)
Impractical application instructions	9.5 (136)	7.9 (90)	6.7 (73)	11.5 (37)	11.9 (32)	11.7 (31)
Drug-Drug- interaction	12.8 (183)	8.5 (97)	9.7 (105)	8.7 (28)	7.8 (21)	8.7 (23)
Contraindication	7.0 (100)	4.2 (48)	5.2 (56)	7.1 (23)	7.4 (20)	5.7 (15)
Duplication of medication	3.3 (48)	1.9 (22)	1.5 (16)	4.0 (13)	4.8 (13)	3.0 (8)
Wrong duration of treatment	16.8 (241)	8.0 (91)	7.8 (85)	23.3 (75)	23.0 (62)	22.0 (58)

*Abbreviationsn*–number of potentially inappropriate medications, t_0_–baseline, t_1_–9 resp. 11 months, t_2_–individual end of treatment, *MAI*–Medication Appropriateness Index

### Primary endpoint: MAI sum and patient scores

The mean MAI sum score amounted to 15.24 ± 12.78 for IG patients at t_0_ and decreased to 7.50 ± 9.54 and 7.64 ± 9.84 at t_1_ and t_2_, respectively. In the CG, the mean MAI sum score amounted to 16.32 ± 13,74 at t_0_ and remained nearly unchanged at t_1_ (16.84 ± 14.25) and t_2_ (16.69 ± 11.29).

The mean MAI sum scores differed significantly between IG and CG at t_1_ and t_2_ (t_1_: −9.34, 95% CI: [−14.31; −4.37], *p* < 0.001, cohen’s d = −0.88, [−1.25; −0.51]; t2: −9.05, 95% CI: [−13.24; −4.87], *p* < 0.001, cohen’s d = −0.89, [−1.27; −0.51]).

In parallel, the mean MAI patient score dropped significantly in the IG from 2.10 ± 1.53 at t_0_ to 0.97 ± 1.13 at t_1_ and 0.91 ± 1.06 at t_2_ (t_1_: −1.39, 95% CI: [−2.00; −0.78], *p* < 0.001, cohen’s d = −1.09, [−1.47; −0.71]; t_2_: −1.41, 95% CI: [−1.98; −0.84], *p* < 0.001, cohen’s d = −1.19, [−1.58; −0.80])).

The correlation in reduction of the MAI sum and patient scores indicate an improved appropriateness of medication therapy and not merely a reduction in the number of medications prescribed. The subsequent Bonferroni adjustment showed that there was only a significant difference in reduction of MAI sum and patient scores between t_0_ and t_1_ as well as t_0_ and t_2,_ but not between t_1_ and t_2_.

**Fig. 1:**
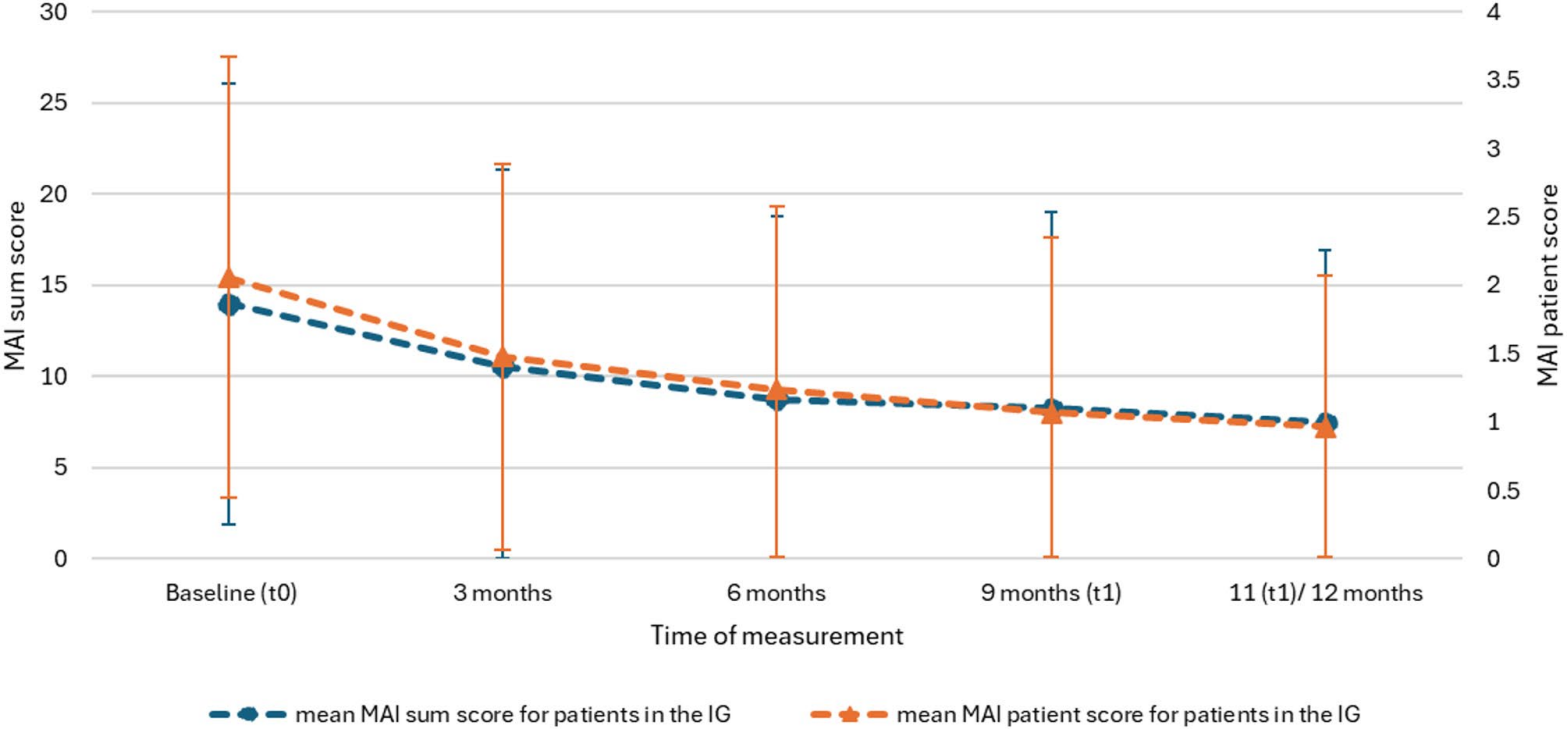
Mean MAI sum and patient scores from baseline to 11/12 months in the IG. (IG–intervention group, MAI–Medication Appropriateness Index)

Analysis of quarterly medication reviews in the IG (see Fig. 1) showed a significant reduction in MAI sum and patient scores after the first medication review at three months; further medication reviews had no significant effect.

### Secondary endpoints: medication appropriateness

Detailed results of PIM analysis regarding the ITT population are shown in Table 4. Among PwD ≥ 65 years in the IG, the mean number of PIM according to the STOPP criteria decreased significantly (*p* < 0.001) from t_0_ to t_1_. However, the decrease in PIM prescriptions for FORTA category D (*p* = 0.401) and the PRISCUS list (*p* = 0.58) revealed to be not significant. Notably, missing medication required for an untreated diagnosis according to the START criteria decreased significantly (*p* < 0.001) from t_0_ to t_1_ in PwD ≥ 65 years in the IG (Table 4).

**Table 4:** Number of PIM prescriptions per patient in the intention-to-treat collective

Secondary endpoint	Intervention Group			Control Group		
	t_0_ (*n* = 194)	t_1_ (*n* = 149)	t_2_ (*n* = 138)	t_0_ (*n* = 45)	t_1_ (*n* = 36)	t_2_ (*n* = 34)
STOPP prescriptions						
M ± SD	1.7 ± 1.65	0.93 ± 1.2	0.91 ± 1.27	1.91 ± 1.72	2 ± 1.84	1.94 ± 1.76
95% CI	[1.46; 1.93]	[0.73; 1.12]	[0.7;1.13]	[1.4; 2.43]	[1.38; 2.62]	[1.33; 2.55]
FORTA D prescriptions						
M ± SD	0.2 ± 0.44	0.15 ± 0.42	0.17 ± 0.4	0.16 ± 0.37	0.19 ± 0.4	0.21 ± 0.41
95% CI	[0.14; 0.26]	[0.09; 0.22]	[0.11; 0.24]	[0.05; 0.27]	[0.06; 0.33]	[0.06; 0.35]
PRISCUS prescriptions						
M ± SD	0.11 ± 0.35	0.09 ± 0.28	0.13 ± 0.38	0.27 ± 0.5	0.25 ± 0.5	0.18 ± 0.39
95% CI	[0.06; 0.16]	[0.04; 0.13]	[0.07; 0.19]	[0.12; 0.42]	[0.08; 0.42]	[0.04; 0.31]
START prescriptions						
M ± SD	0.85 ± 0.98	0.58 ± 0.85	0.49 ± 0.74	0.53 ± 0.76	0.61 ± 0.8	0.62 ± 0.82
95% CI	[0.71; 0.98]	[0.45; 0.72]	[0.36;0.61]	[0.31; 0.76]	[0.34; 0.88]	[0.33; 0.9]

*Abbreviationsn*–number of patients aged ≥ 65 years, t_0_–baseline, t_1_–9 resp. 11 months, t_2_–individual end of treatment, *M*–mean value, *SD*–standard deviation, *CI* – confidence interval

Pharmaceutical recommendations regarding deprescribing (IG only) led to a significant reduction in prescriptions of high-risk drugs (t_0_ = 0.74 [95% CI: 0.6; 0.88], t_1_ = 0.51 [95% CI: 0.37; 0.65]), *p* = 0.022). Regarding high-risk medication, ceasing antipsychotic treatment was the most common pharmaceutical recommendation at all time points, followed by discontinuation of anticholinergics at t_0_ and discontinuation of proton pump inhibitors at t_1_ and t_2_. The greatest reductions in inappropriate prescribing of high-risk drugs was seen for anticholinergics from t_0_ to t_1_ (−7.6%) and for antipsychotics, intended to treat behavioural and psychological symptoms of dementia, from t_1_ to t_2_ (−10.9%).

**Fig. 2:**
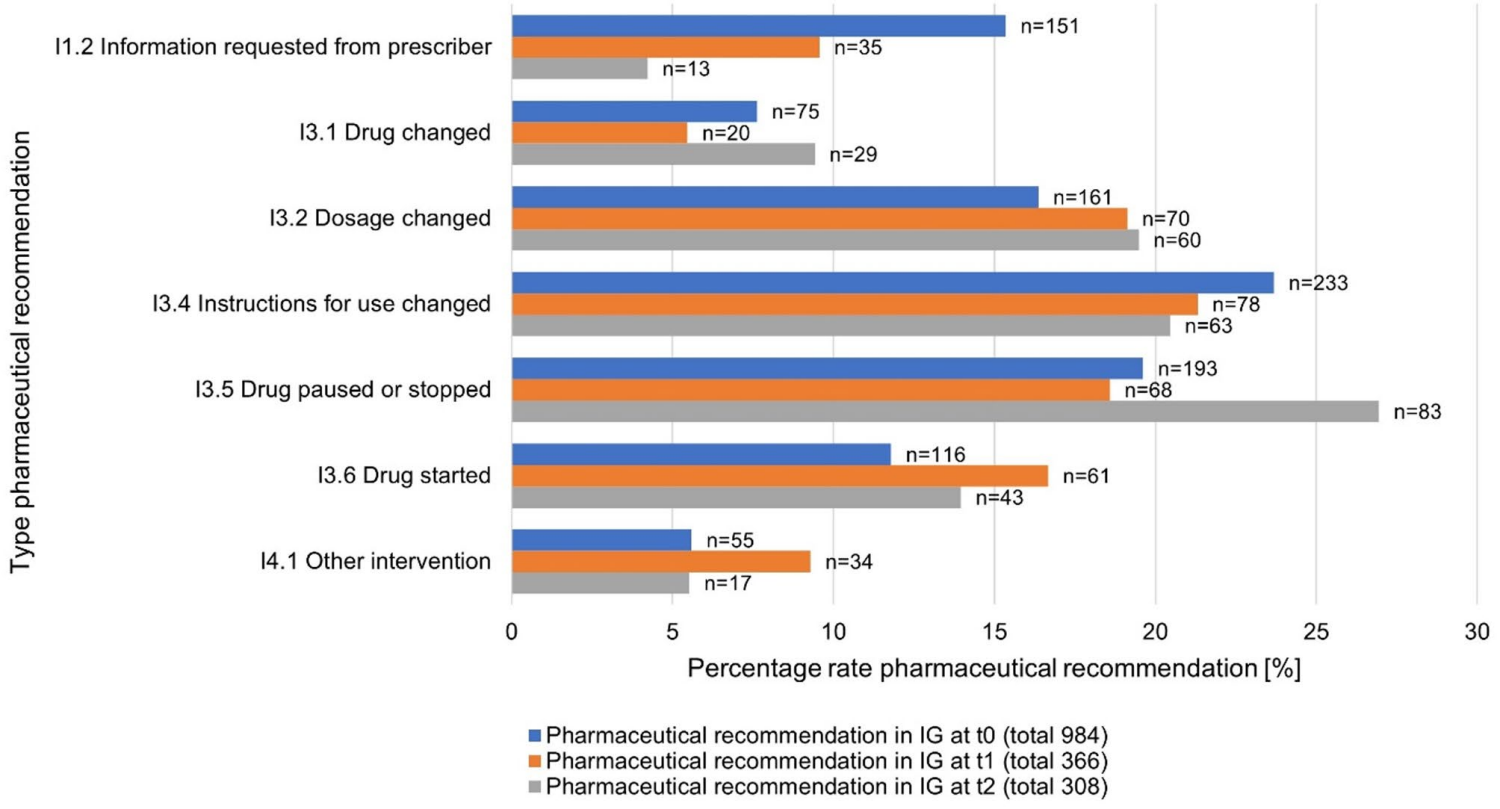
Type, rate, and frequency of pharmaceutical recommendation given for patients in the IG. (n–frequency of pharmaceutical recommendation, IG-intervention group, t_0_–baseline, t_1_–9 resp. 11 months, t_2_–individual end of treatment)

Pharmaceutical recommendations significantly (*p* < 0.001) reduced the number of DRPs in the IG from to 4.97 [95% CI: 4.47; 5.47] at t_0_ to 2.39 [95% CI: 1.94; 2.84]) at t_1_. Figure 2 shows the type and frequency of pharmaceutical recommendation according to PCNE version 9.1 at t_0_, t_1_, t_2_ given to the GP via the electronic patient record. On the level of drug classes (ATC 2nd level), antithrombotic agents were most frequently aligned to DRP at time point t_0,_ while at time point t_1_ and t_2_, DRPs were most frequently related to psycholeptics. On the level of active substances (ATC 5th level), pantoprazole caused by far the highest number of DRPs at each time point.

## Discussion

Results of the study revealed that pharmacist-led medication reviews are useful to increase appropriate prescribing and medication safety in PwD. This finding is consistent with previous studies on the impact of medication reviews in PwD performed by clinical pharmacists in different outpatient settings [34, 35]. According to the status analysis of the participating PwD, medication therapy in both groups (IG and CG) was characterised by a high prevalence of poor prescribing, especially in cases of polypharmacy. Polypharmacy per se could not be reduced through pharmaceutical interventions. However, the number of prescribed medications does not necessarily reflect medication appropriateness. The primary endpoint, i.e. impact of pharmacist-led medication reviews on medication appropriateness, was proven in the IG by a significant decrease of the MAI. The initial medication review of the responsible pharmacist and prompt realisation of the resulting pharmaceutical recommendations by the responsible GP reduced the MAI sum and MAI patient scores significantly. Ongoing quarterly medication reviews did not result in further, significant improvement of medication appropriateness. This finding can be related to short intervals between the medication reviews and limited progress of dementia and co-morbidities of the patients. The MAI revealed to be feasible for the detection of inappropriate medication in the study population. Analysis of the primary endpoint showed equal results for the per protocol (PP) population (153 patients) and the ITT population (198 patients). Therefore, they were considered equivalent for all further analyses.

Of note, the rate of prescribed PIMs identified by the FORTA and PRISCUS list in PwD above 65 years also decreased. Missing significance of the decrease is explained by an overall low rate of PIM prescriptions. Tools such as the FORTA and PRISCUS list and the STOPP criteria revealed to be useful for identifying PIM for geriatric PwD. Results of the different tools were similar. Analysis of the drug groups and substances causing DRP showed that medication reviews should consider all medications taken by the patient instead of being limited to certain drugs or drug groups.

In the primary care setting, potentially inadequate medication is often not identified since local pharmacists do not have comprehensive information on diagnosis, laboratory parameters and cognitive status when performing medication reviews in PwD. Implementation of cooperative medication management of GPs and pharmacists should be promoted, especially when considering the improved MAI in the current study. In Germany, missing interdisciplinary medication management is partly due to the lack of electronic patient records and digitalised communication in primary care [36]. Positive effects of collaborative networks and electronic patient records on improving quality and safety of medication therapy are obvious [37, 38]. Recently, collaborative medication management was demanded to improve dementia care [39]. Pharmacist-led systematic medication reviews and cooperation with GPs should be further investigated in patient studies and implemented in routine primary care. The need for regularly practised medication reviews in PwD is justified by the diagnosis, co-morbidities, polypharmacy and associated DRP as well as proven benefit.

Nevertheless, the study has several limitations. First, the medication reviews were based on electronic patient records filled by the GP and the case manager. A bias due to incorrect or missing documentation cannot be ruled out. Moreover, changes of inappropriate prescribing rates can also be related to missing information in the electronic patient records. As documentation of the acceptance of a pharmaceutical recommendation was not mandatory, the acceptance status of pharmaceutical recommendations was mainly evaluated by identification of modified prescribing at the following observation time point. Consistently, the impact of pharmaceutical recommendations decreased over time because the number of remaining DRPs decreased.

Second, another limitation is the small number of cases in the study and the difference in sample size between the IG and the CG. The targeted number of 120 GPs, each one recruiting 13–15 PwD was not achieved in the DemStepCare study. As the sample size was smaller than planned, reliability of extrapolated results is limited. In addition, the lack of an interface for communication between the electronic patient record used in the study and the respective physician office software resulted in a considerable amount of work for the participating GP.

Results of the DemStepCare study show that there is a need for medication reviews in PwD. All tools used revealed to be suitable for detecting inappropriate medication thereby improving quality and safety of medication in PwD. Pharmaceutical medication reviews, corresponding to those practiced in the DemStepCare study, can be beneficially implemented in routine care, provided that electronic patient records are available.

## Conclusion

A single, initial pharmacist-led medication review improved the medication appropriateness and safety in PwD over an extended period, indicated by significantly reduced MAI sum and patient scores. However, as additional medications are prescribed as dementia progresses, regular pharmacist-led medication reviews are recommended in PwDs. Electronic patient records and digital networks are needed to increase efficiency of pharmacist-led medication reviews in PwD.

## Supplementary Information

Supplementary Material 1.

## Data Availability

No datasets were generated or analysed during the current study.

## Abbreviations

CG: Control group
CI: Confidence interval
DRP: Drug related problems
FORTA: Fit for the aged
GP: General practitioner
IG: Intervention group
ITT: Intention to treat
MAI: Medication appropriateness index
PCNE: Pharmaceutical care network europe
PIM: Potentially inappropriate medication
PP: Per protocol
PS: Patient score
PwD: Patients with dementia
SS: Sum score
START: Screening tool to alert to right treatment
STOPP: Screening tool for older persons prescription
