# Supplementary material for: Effect of pharmacist-led medication reviews on appropriateness of prescribing in patients with dementia – results from the cluster randomized controlled “DemStepCare” study
Source: BMC Geriatr. 2025 Nov 4;25:842. doi: 10.1186/s12877-025-06565-6 (PMC12584325; doi:10.1186/s12877-025-06565-6)
Supplement: Supplementary file 1 — Supplementary Material 1. [file 12877_2025_6565_MOESM1_ESM.docx]

Supplement 1

Adapted from Hanlon et al (28, 29):

| Question | Group | Weight |
| --- | --- | --- |
| 1. Is there an indication for the drug? | A | 3 |
| 1. Is the medication effective for the condition | A | 3 |
| 1. Is the dosage correct? | B | 2 |
| 1. Are the directions correct? | B | 2 |
| 1. Are the directions practical? | C | 1 |
| 1. Are there clinically significant drug-drug interactions? | B | 2 |
| 1. Are there clinically significant drug-disease/condition interactions? | B | 2 |
| 1. Is there unnecessary duplication with other drug(s)? | C | 1 |
| 1. Is the duration of therapy acceptable? | C | 1 |
| 1. Is this drug the least expensive alternative compared to others of equal utility? | C | 1 |
